# Predictive Value of Echocardiographic Pulmonary to Left Atrial Ratio for In-Hospital Death in Patients with COVID-19

**DOI:** 10.3390/diagnostics13020224

**Published:** 2023-01-07

**Authors:** Giulia Renda, Marco G. Mennuni, Giovanni Pizzoferrato, Daniele Esposto, Angela Alberani, Simona De Vecchi, Anna Degiovanni, Ailia Giubertoni, Enrico Guido Spinoni, Leonardo Grisafi, Emanuele Sagazio, Claudio Ucciferri, Katia Falasca, Jacopo Vecchiet, Sabina Gallina, Giuseppe Patti

**Affiliations:** 1 Institute of Cardiology, Department of Neuroscience, Imaging and Clinical Sciences, G. d’Annunzio University of Chieti-Pescara, 66100 Chieti, Italy; 2 SS. Annunziata Hospital of Chieti, 66100 Chieti, Italy; 3 Maggiore della Carità Hospital, 28100 Novara, Italy; 4 Translational Medicine Department, University of Eastern Piedmont, 28100 Novara, Italy; 5 Clinic of Infectious Diseases, Department of Medicine and Science of Aging, G. d’Annunzio University of Chieti-Pescara, 66100 Chieti, Italy

**Keywords:** ePLAR, pulmonary embolism, trans-pulmonary pressure gradient, COVID-19, in-hospital mortality

## Abstract

Background: Echocardiographic Pulmonary to Left Atrial Ratio (ePLAR) represents an accurate and sensitive non-invasive tool to estimate the trans-pulmonary gradient. The prognostic value of ePLAR in hospitalized patients with COVID-19 remains unknown. We aimed to investigate the predictive value of ePLAR on in-hospital mortality in patients with COVID-19. Methods: One hundred consecutive patients admitted to two Italian institutions for COVID-19 undergoing early (<24 h) echocardiographic examination were included; ePLAR was determined from the maximum tricuspid regurgitation continuous wave Doppler velocity (m/s) divided by the transmitral E-wave: septal mitral annular Doppler Tissue Imaging e′-wave ratio (TRVmax/E:e′). The primary outcome measure was in-hospital death. Results: patients who died during hospitalization had at baseline a higher prevalence of tricuspid regurgitation, higher ePLAR, right-side pressures, lower Tricuspid Annular Plane Systolic Excursion (TAPSE)/ systolic Pulmonary Artery Pressure (sPAP) ratio and reduced inferior vena cava collapse than survivors. Patients with ePLAR > 0.28 m/s at baseline showed non-significant but markedly increased in-hospital mortality compared to those having ePLAR ≤ 0.28 m/s (27% vs. 10.8%, *p* = 0.055). Multivariate Cox regression showed that an ePLAR > 0.28 m/s was independently associated with an increased risk of death (HR 5.07, 95% CI 1.04–24.50, *p* = 0.043), particularly when associated with increased sPAP (*p* for interaction = 0.043). Conclusions: A high ePLAR value at baseline predicts in-hospital death in patients with COVID-19, especially in those with elevated pulmonary arterial pressure. These results support an early ePLAR assessment in patients admitted for COVID-19 to identify those at higher risk and potentially guide strategies of diagnosis and care.

## 1. Introduction

Coronavirus Disease-2019 (COVID-19), caused by Severe Acute Respiratory Syndrome CoronaVirus-2 (SARS-CoV-2) infection, continues to cause considerable morbidity and mortality worldwide [1]. Patients hospitalized for COVID-19 usually present with a respiratory syndrome and frequently suffer from macrovascular thrombotic complications impairing early survival [1,2,3]. Indeed, autoptic data also indicated a diffuse microvascular thrombosis in the lungs of patients who died from COVID-19 [4,5]. In this setting, a pro-thrombotic milieu, due to “cytokine storm”, endothelial damage and inflammation, may favor pulmonary vascular obstruction and predispose patients to an acute afterload increase, causing pulmonary hypertension and right ventricle dysfunction [6]. Notably, right ventricle dysfunction and pulmonary hypertension, assessed by transthoracic echocardiography, have been associated with higher mortality in COVID-19 [6,7]. The absence of abnormalities in traditional echocardiographic parameters evaluating right ventricle function or pulmonary hypertension does not definitively exclude pulmonary thrombotic complications, particularly in clinically stable patients with normal or slightly increased pulmonary pressures [8]. On the other hand, a right ventricle dysfunction may also be due to pre-existing right-heart pathologies, even in the absence of pulmonary vasculature abnormalities [9]. Echocardiographic evidence of right-heart dysfunction is routinely used in clinical practice in cases of suspected pulmonary hypertension [10] and has an established prognostic value in the acute [11] and long-term assessment [12] of pulmonary embolism, a condition well-known to be associated with COVID-19 [13]. Several of the echocardiographic parameters used to explore right-heart dysfunction reflect pre-capillary obstruction in the pulmonary vascular bed and a subsequent trans-pulmonary pressure gradient (the pressure gradient between pulmonary artery and left atrium). The ePLAR (echocardiographic Pulmonary to Left Atrial Ratio) is a novel parameter validated as a non-invasive substitute for trans-pulmonary gradient [14];ePLAR assesses the relationship between right ventricular systolic pressure and left atrial pressure via the formula ePLAR (m/s) = TRV (tricuspid regurgitation velocity) max (m/s)/mitral E/e’ [14]. It may be able to differentiate pre-capillary and post-capillary pulmonary hypertension. A validation study showed that an ePLAR value >0.28 m/s is indicative of a high transpulmonary gradient with a higher diagnostic accuracy than standard parameters such as TRV max and TAPSE (Tricuspid Annular Plane Systolic Excursion) [15]. Therefore, increased ePLAR values, even in patients with sub-massive acute pulmonary embolism and normal/near normal estimated pulmonary pressures, may suggest an increased trans-pulmonary gradient with pre-capillary obstruction to the pulmonary flow [15].

To the best of our knowledge, there are no data on ePLAR assessment in hospitalized patients with COVID-19. These patients often exhibit preserved conventional echocardiographic parameters, challenging the risk stratification. In such a context, the value of ePLAR could be of additional significance. Accordingly, the present study was aimed to investigate the prognostic value of ePLAR at baseline on early mortality in patients hospitalized for COVID-19.

## 2. Methods

### 2.1. Study Population and Data Collection

This is a prospective, observational study performed in two centers: Maggiore della Carità Hospital in Novara, Italy, and SS. Annunziata Hospital in Chieti, Italy. Consecutive patients hospitalized for SARS-CoV-2 infection from 1 March 2021, through 31 May 2021, receiving an early (<24 h) echocardiographic evaluation upon admission were enrolled. The diagnosis of SARS-CoV-2 infection was confirmed by reverse-transcriptase polymerase chain reaction on a nasopharyngeal swab. A case report was created using an electronic data capture software, where individual data obtained after the revision of clinical records were entered. A unique pseudonymized code was assigned to each participant. Individual data included physical characteristics, medical history, cardiovascular risks factors, laboratory findings, medical treatments and clinical events during in-hospital stay (acute myocardial infarction, acute heart failure, acute pericarditis, atrio-ventricular block, sustained ventricular tachycardia, ventricular fibrillation, need for intensive care unit, pulmonary embolism, deep vein thrombosis, TIA or ischemic stroke, septic shock, acute renal failure, in-hospital death, major adverse cardiovascular events (MACE) in term of death, acute myocardial infarction, transient ischemic attack, stroke or venous thromboembolism).

Patients were enrolled regardless of the severity of COVID-19 clinical presentation and of in-hospital therapies for the SARS-CoV-2 infection. However, patients requiring early invasive ventilation before transthoracic echocardiography (TTE) were excluded to avoid any confounding for echocardiographic parameters. Patients with COVID-19 who died after admission but before TTE examination were also excluded. The study protocol was approved by the institutional ethical committee (IRB code CE 97/20) and conducted strictly according to the principles of the Declaration of Helsinki. The authors have full access to all the data in the study and take responsibility for its integrity and the data analysis. 

### 2.2. Echocardiography Assessment 

TTE examination was performed <24 h after admission, and echocardiographic parameters were recorded and measured according to recommendations of international guidelines [16]. In particular, TRV max was measured by identifying tricuspid regurgitation at color Doppler imaging and designing the contour of the jet at continuous-wave Doppler imaging. Right atrial pressure (RAP_echo_) was assessed by measuring inferior vena cava (IVC) diameter and its variations during the respiratory cycle, including a brief sniff to elicit the inspiratory response. Systolic pulmonary arterial pressure (sPAP) was estimated by adding RAP_echo_ to the maximal systolic pressure gradient from tricuspid regurgitation velocity (TRV). TAPSE was calculated in the RV free wall as perpendicular to the lateral tricuspid annulus from the apical 4-chamber view using an M-mode cursor tracing. Left-heart diastolic filling was assessed using pulsed wave Doppler at the mitral tips according to the international recommendations [17]. Mitral annular Doppler Tissue Imaging (DTI) velocities were assessed in the annulus (septal and lateral); ePLAR was measured as described above. All measurements were averaged over three beats in sinus rhythm and five nonconsecutive beats with cycle lengths within 10% to 20% of the average heart rate in atrial fibrillation.

For the purpose of the study, patients were divided according to the value of ePLAR at baseline in those with high (>0.28 m/s) and low (≤0.28 m/s) ePLAR [15]. Patients without fully interpretable images were excluded. Reproducibility of all echocardiographic measures was confirmed by two different physicians to minimize inter-individual variability. Following a recent practical guideline [18], the reproducibility was blind tested in a random sample of 15 patients (Supplementary Figure S1).

Primary outcome was the incidence of all-cause death during in-hospital stay in patients with high vs. low ePLAR values.

### 2.3. Statistical Analysis

Continuous variables are indicated as mean ± standard deviation and were analyzed by *t*-test or Wilcoxon test, as appropriate. Categorical variables are reported as frequencies (percentage) and were analyzed by the Chi-square test. Thirty-day survival rates were estimated using the Kaplan Meier method and presented as survival curves in patients with high (>0.28 m/s) vs. low (≤0.28 m/s) ePLAR at baseline. The log-rank test was utilized to assess differences in 30-day survival between ePLAR subgroups. Receiver Operating Characteristic (ROC) analysis was performed to describe the association between high ePLAR value and in-hospital death. The Cox proportional hazards model was used to estimate the independent association between high ePLAR value at baseline and subsequent mortality.

The multivariable model included as covariates demographic factors, comorbidities and other echocardiographic parameters. Hazard ratio (HR) and 95% confidence interval (CI) were calculated. A forward stepwise approach to identify independent predictors was used. In particular, variables with a *p* value < 0.10 at univariate analysis were entered into the multivariate model, and clinically meaningful covariates were forced into the model irrespective of their *p* values (i.e., gender, arterial hypertension, diabetes mellitus, atrial fibrillation, severe ARDS at presentation left ventricular ejection fraction, and TAPSE).

Finally, an interaction test was performed to evaluate the association of ePLAR values with the risk of death across different sPAP levels.

All calculations were performed using statistical software STATA 16.0 (StataCorp, LP, College Station, TX, USA). All tests were two-sided, and a *p*-value < 0.05 was considered statistically significant.

## 3. Results

**Figure 1:**
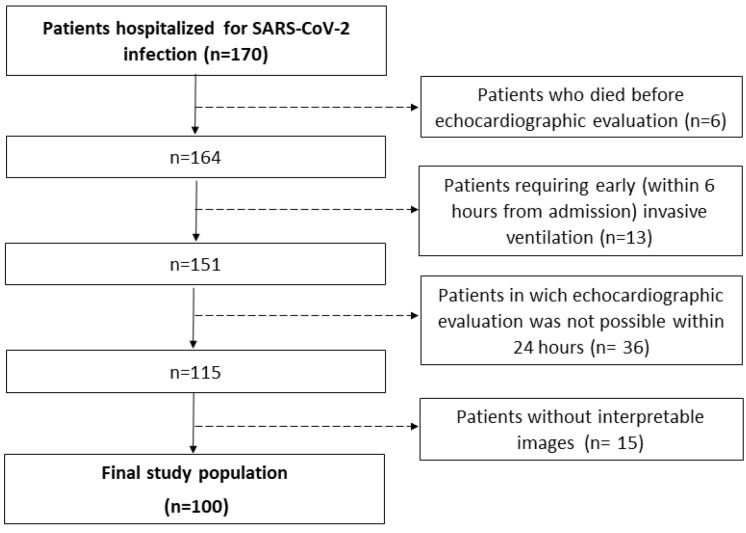
Flow diagram showing how the final study population was obtained.

From 1 March 2021 through 31 May 2021, a total of 170 patients were admitted for SARS-CoV-2 infection at the two institutions. A total of 6 patients died early after hospitalization, 13 required early invasive ventilation and 36 could not receive an echocardiographic evaluation <24 h. Therefore, 115 patients underwent early echocardiography, with 15 of them having no interpretable images. Thus, 100 patients were enrolled in the study (Figure 1).

The main demographic/clinical characteristics in the overall population and according to ePLAR value at baseline are reported in Table 1. The mean age was 65 years, and the prevalence of female gender was 38%. A total of 63 patients had a high ePLAR value (>0.28 m/s) and 37 a low ePLAR value (≤0.28 m/s). Compared to patients having low ePLAR, those with high ePLAR presented higher body weight, higher body surface area, non-significant higher proportion of men, lower prevalence of atrial fibrillation, lower chronic obstructive pulmonary disease and reduced PaO_2_/FiO_2_ at presentation. The latter value may not be of clinical significance because both values belong to the same clinical risk class (i.e., mild ARDS).

Echocardiographic parameters are indicated in Table 2. Compared to patients with low ePLAR, those with high ePLAR had reduced left ventricular size, left atrial volume, E wave velocity, mitral septal e’ velocity, E/e’ and TAPSE/sPAP, as well as more elevated left ventricular ejection fraction, TAPSE, E/A ratio, TRV, tricuspid s’ velocity, mitral lateral e’ velocity and mitral medium e’ velocity.

In-hospital adverse events are reported in Table 3. During in-hospital stay, 21 patients died. Non-survivors were older and had a higher prevalence of diabetes mellitus, chronic renal failure, and chronic liver disease, as well as severe ARDS (PaO_2_/FiO_2_ < 100) and lower arterial oxygen saturation at presentation compared to survivors (Supplementary Table S1). Regarding echocardiographic parameters (Table 4), non-survivor patients showed increased prevalence of tricuspid regurgitation and mitral regurgitation, reduced rate of inferior vena cava collapse, higher tricuspid regurgitation velocity, sPAP, right ventricular systolic pressure and A wave velocity, lower mitral medium e’ velocity, TAPSE/sPAP and left ventricular end-diastolic diameter.

**Figure 2:**
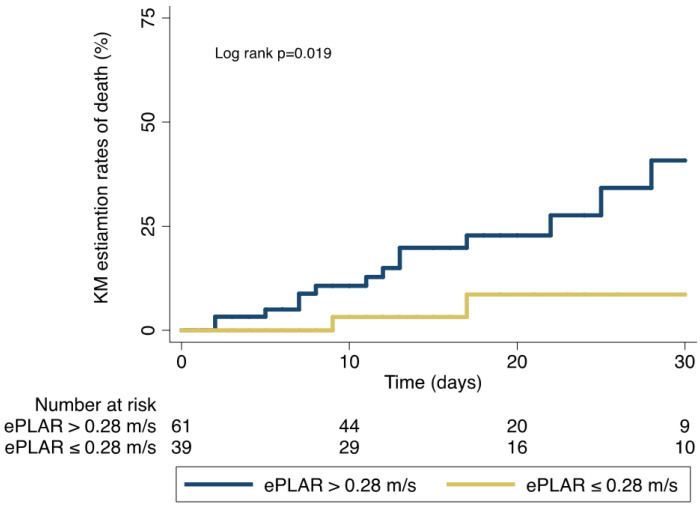
30-day all-cause death in patients with COVID-19 with high vs. low ePLAR. Kaplan–Meier curves show the estimation rates of death in patients with high vs. low ePLAR value at baseline (cut-off 0.28 m/s); ePLAR = echocardiographic Pulmonary to Left Atrial Ratio.

In-hospital mortality was 27% (*n* = 17) in patients with high ePLAR vs. 10.8% (*n* = 4) in those with low ePLAR (*p* = 0.05). Figure 2 shows Kaplan–Meier curves for the estimate of 30-day all-cause death in patients with high vs. low ePLAR values at baseline (log rank *p* = 0.019). At ROC analysis, the area under the curve for in-hospital death with ePLAR > 0.28 m/s was 0.52 (95% CI 0.40–0.63). For an ePLAR value > 28 m/s, sensitivity for in-hospital death was 81%, specificity 42%, positive predictive value 27% and negative predictive value was 89%. 

**Figure 3:**
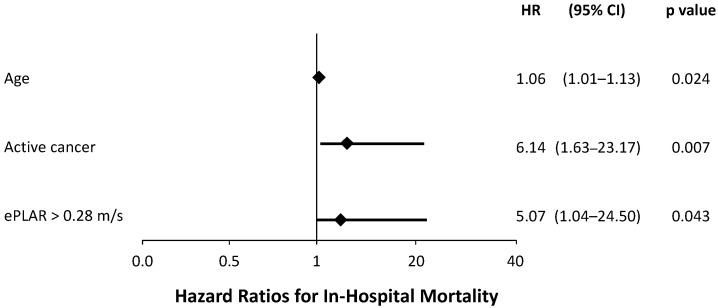
Multivariate Cox regression analysis for independent predictors of in-hospital death. A high ePLAR value (>0.28 m/s) at baseline is an independent predictor of in-hospital mortality. Other independent predictors are age and active cancer; ePLAR = echocardiographic Pulmonary to Left Atrial Ratio.

Univariate Cox proportional hazard regression identified age (HR 1.07, *p* = 0.003), body surface area (HR 0.08, *p* = 0.055), active cancer (HR 5.59, *p* = 0.003), chronic renal failure (HR 6.35, *p* < 0.001), chronic liver disease (HR 8.03, *p* = 0.007), P/F at presentation (HR 0.99, *p* = 0.096), LVEF (HR 0.99, *p* = 0.891), A wave velocity (HR 1.02, *p* = 0.042), Mitral medium e’ velocity (HR 0.78, *p* = 0.070), sPAP (HR 1.03, *p* = 0.061) and ePLAR (HR 4.50, *p* = 0.047) associated with an increased risk of death. Multivariate Cox model identified a high ePLAR value at baseline as an independent predictor of in-hospital mortality (HR 5.07, 95% CI 1.04–24.50, *p* = 0.043) (Figure 3). Other independent predictors of reduced survival were age (HR 1.06, 95% CI 1.01–1.13, *p* = 0.024) and active cancer (HR 6.14, 95% CI 1.63–23.17, *p* = 0.007).

**Figure 4:**
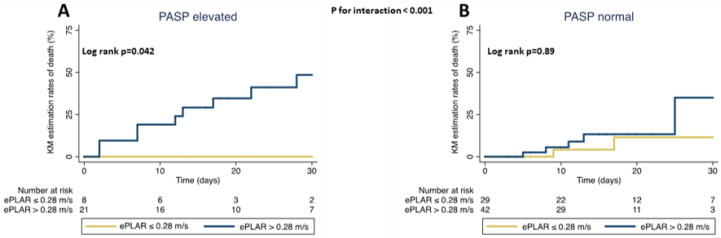
30-day all-cause death in patients with COVID-19 with high vs. low ePLAR, according to sPAP values. Kaplan–Meier curves show the estimation rates of death with high vs. low ePLAR value at baseline (cut-off 0.28 m/s) in subgroups of patients with elevated (panel **A**) and normal (panel **B**) sPAP; ePLAR = echocardiographic Pulmonary to Left Atrial Ratio; sPAP = systolic pulmonary artery pressure.

A high ePLAR value was associated with increased mortality mainly in the subgroup of patients with elevated (>35 mmHg) sPAP (*p* for interaction = 0.043), as estimated by trans-thoracic echocardiography (Kaplan–Meier estimated survival at 30 days with high vs. low ePLAR value: 51% vs. 100%, log rank *p* = 0.042) (Figure 4, panel A). Conversely, the association between high ePLAR and lower survival was not significant in patients with sPAP ≤ 35 mmHg (KM estimated survival at 30 days with high vs. low ePLAR value: 65% vs. 88%, log rank *p* = 0.89; *p* for interaction < 0.001) (Figure 4, panel B). At ROC analysis, the area under the curve for in-hospital death with ePLAR > 0.28 m/s coupled with sPAP > 35 mmHg was 0.73 (95% CI 0.61–0.84). For an ePLAR value > 28 m/s coupled with sPAP > 35 mmHg, sensitivity for in-hospital death was 57%, specificity 89%, positive predictive value 58% and negative predictive value was 89%.

## 4. Discussion

Our prospective study indicates that a high ePLAR value, an echocardiographic index of trans-pulmonary pressure gradient, when assessed early after the admission, is significantly associated with in-hospital mortality in patients with COVID-19. 

The coagulation system plays a pivotal role in the pathogenesis of COVID-19 complications [1,2,3]; this has been confirmed by recent demonstrations from autoptic studies reporting in this setting both micro and macrovascular lung thrombosis [4,5]. Consequently, right-heart disease due to pulmonary thrombosis may have a significant impact on clinical outcome and overall prognosis in patients with COVID-19. Moreover, patients hospitalized for COVID-19 often present a high occurrence of left ventricular dysfunction (ranging from 10% to 30 % of cases), with equally higher morbidity and mortality [19,20,21]. Furthermore, COVID-19-related cardiomyopathies and lung complications share a high prevalence of factors impairing short-term and long-term prognosis, such as older age, obesity, arterial hypertension, diabetes mellitus, atrial fibrillation and chronic obstructive pulmonary disease [22,23,24]. Considering this evidence, in patients admitted for COVID-19, it remains sometimes difficult to make a differential diagnosis between increased post- vs. pre-capillary pulmonary pressures. Recently, ePLAR has been validated as a non-invasive surrogate for trans-pulmonary pressure gradient between the pulmonary artery and the left atrium. In particular, it has demonstrated a sensitivity for pre-capillary pulmonary obstruction higher than traditional echocardiographic measures of right ventricular pressure and function, even in the absence of markedly increased pulmonary pressures or right ventricular dysfunction [15].

In this multicenter investigation, we first found that ePLAR > 0.28 m/s at baseline was a significant predictor of in-hospital death in patients admitted for COVID-19. The increase in mortality in patients with increased ePLAR was 5-fold at multivariate analysis and independent of potential confounders. Importantly, the discriminative power of an ePLAR value ≤ 28 m/s for excluding early mortality was very high, with a negative predictive value of 89%; this makes the ePLAR suitable for an early screening of in-hospital admitted patients. On the other hand, the low positive predictive value can be explained by different causes and pathogenetic mechanisms of all-cause mortality in this population. 

Notably, in a previous investigation, individuals with ePLAR > 0.28 m/s had an elevated trans-pulmonary gradient related to a diffuse thrombosis of the pulmonary vascular bed, even when small vessels were involved and in the absence of markedly increased pulmonary artery pressures [15]. In our population, patients with ePLAR > 0.28 m/s had a higher (but normal) TRV and a reduced E/e’ vs. those with lower ePLAR values; this corroborates the presence of a pre-capillary pulmonary obstruction. However, compared to patients with low ePLAR, those having a high ePLAR value at baseline presented on average similar right ventricular dimensions and pulmonary pressures and even more elevated TAPSE and tricuspid s’ velocity. Interestingly, in the population with high ePLAR there were no cases of macro pulmonary embolism. This confirms the value of ePLAR in this population as a marker of increased trans-pulmonary gradient secondary to pulmonary microvascular obstruction. These findings might indicate that in patients with pre-capillary pulmonary obstruction, ePLAR modifications precede the impairment of traditional echocardiographic parameters related to right ventricular function and anticipate such parameters in predicting a poorer outcome. Accordingly, patients with higher ePLAR also had a decreased TAPSE/sPAP ratio. Indeed, in patients with pre-capillary pulmonary hypertension, this feature typically indicates a compromised hemodynamic status and a worse prognosis [25]. On the other hand, patients with ePLAR ≤ 0.28 m/s had frequent more left-side heart impairment, as indicated by larger left ventricular dimensions, increased left atrial volume, higher E wave velocity, reduced left ventricular ejection fraction and more frequent occurrence of AF. Of note, we found that a high ePLAR value predicted all-cause death mainly in the subgroup with elevated pulmonary artery systolic pressure (>35 mmHg), e.g., when the expected trans-pulmonary pressure gradient is more pronounced. Here, the AUC for in-hospital mortality was 0.73 and specificity raised to 89%.

Our results support an extensive ePLAR assessment in patients hospitalized for COVID-19 in whom the detection of a higher value may support a strategy of targeted multimodality imaging to detect thrombotic complications in the pulmonary vascular bed. An early detection of these pulmonary complications may be critical in driving strategies of more intense anticoagulation. As a matter of fact, evidence from available investigations comparing different heparin doses in COVID-19 yielded conflicting results [26,27,28]. Randomized trials indicated a signal towards increased survival with therapeutic vs. prophylactic doses of low molecular weight heparin among moderately ill patients [27]. In contrast, no benefit was observed with therapeutic heparin dosages among critically ill patients [28]. A possible explanation of the latter finding is that underlying thrombotic and inflammatory damage was too advanced to be influenced by higher doses of heparin. 

We observed an overall 21% in-hospital mortality, and, consistent with other studies [22,23,24], non-survivor patients were older and had more comorbidities than survivors, as indicated by a higher prevalence of diabetes mellitus, chronic renal failure and chronic liver disease. Furthermore, as expected, they more frequently had severe acute respiratory distress syndrome and reduced arterial oxygen saturation at emergency department presentation. This more severe respiratory syndrome can be at least in part due to microvascular thrombosis in the pulmonary bed [1,2], explaining the higher prevalence of tricuspid regurgitation, reduced rates of inferior vena cava collapse, higher sPAP and right ventricular systolic pressure and lower TAPSE/sPAP ratio in non-survivors compared to survivors.

Our investigation has limitations inherent to all observational studies, e.g., inclusion bias, treatment bias, residual confounding and a possible competitive risk bias. First of all, we may have introduced an inclusion bias because we collected data from 100 of the 170 patients hospitalized for COVID-19 in the observational period: excluding patients who early died (6 patients) and those requiring early invasive ventilation (13 patients). We obtained echocardiograms from 76% (66% with interpretable images) of the remaining population. In the other 24%, we could not perform the exam within 24 h due to logistic and clinical reasons. Furthermore, laboratory and invasive hemodynamics data were not available; data collection on medical history and comorbidities was often based on patients’ reports and therefore is potentially biased. Moreover, our findings are highly consistent with the hypothesis that patients with high ePLAR values suffered from diffuse, microvascular thrombosis in the lung, leading to impairment of pulmonary function and increased mortality; however, a documentation on that was not available, and on the other hand, the specificity of ePLAR for microvascular pulmonary thrombosis was not extensively addressed in previous studies. Finally, we were not able to explore the relationship between ePLAR and mortality risk over the long-term in COVID-19; future investigations focused on this issue are welcome. Future research should investigate whether the course over time of these echocardiographic parameters, possible in the form of serial measurements, carries additional prognostic value over a single assessment at baseline in hospitalized patients with COVID-19, as already observed to be the case in laboratory parameters [29]. Another issue to be clarified is whether the transformation from continuous to dichotomous variable of the value of ePLAR could cause a loss of information of this diagnostic tool.

## 5. Conclusions

The predictive value of ePLAR observed in our study might find a clinical application to identify patients hospitalized for COVID-19 at higher risk of death early and guide diagnostic and therapeutic approaches. Indeed, owing to the association between ePLAR and pre-capillary pulmonary obstruction, such a parameter could be helpful to select those patient candidates worthy of stricter diagnostic/imaging testing and “more aggressive” antithrombotic therapies. However, our results are hypothesis generating and merit confirmation in specific, prospective studies on larger populations.

## Figures and Tables

**Table 1:** Demographic/clinical characteristics of patients with COVID-19, in the overall population and according to ePLAR values at baseline.

	Overall *n* = 100	High ePLAR *n* = 63	Low ePLAR *n* = 37	*p* Value
Age (years)	64.9 ± 15.4	62.8 ± 15.3	68.5 ± 15.2	0.075
Gender female	38 (38.0)	21 (33.3)	17 (46.0)	0.210
Body weight (Kg)	80.9 ± 17.8	84.3 ± 18.5	75.1 ± 15.0	**0.012**
BMI (Kg/m^2^)	28.2 ± 5.2	28.9 ± 5.3	26.9 ± 4.8	0.072
BSA (m^2^)	1.9 ± 0.2	2.0 ± 0.2	1.8 ± 0.2	**0.008**
Arterial Hypertension	64	40 (63.5)	24 (64.9)	0.890
Diabetes mellitus	28	18 (28.6)	10 (27.0)	0.868
Smoking	16	10 (15.9)	6 (16.2)	0.964
Ischemic Heart Disease	13	6 (9.5)	7 (18.9)	0.177
Non-Ischemic Heart Disease	14	7 (11.1)	7 (18.9)	0.277
Previous PCI	10	5 (7.9)	5 (13.5)	0.369
Previous CABG	5	3 (4.8)	2 (5.4)	0.887
Atrial Fibrillation	15	6 (9.5)	9 (24.3)	**0.045**
COPD	13	5 (7.9)	8 (21.6)	**0.049**
Active Cancer	9	5 (7.9)	4 (10.8)	0.628
History of cancer	11	7 (11.1)	4 (10.8)	0.963
Autoimmune Disease	10	4 (6.4)	6 (16.2)	0.112
Chronic renal failure	15	11 (17.5)	4 (10.8)	0.369
Chronic liver disease	2	2 (3.2)	0 (0)	0.274
Severe ARDS at ED presentation	36	27 (42.9)	9 (24.3)	0.062
Systolic Blood Pressure (mmHg)	131.0 ± 19.1	131.5 ± 18.5	130.1 ± 20.2	0.717
Diastolic Blood Pressure (mmHg)	75.3 ± 12.6	75.0 ± 11.5	75.9 ± 14.4	0.705
Heart Rate (bpm)	86.4 ± 17.3	86.5 ± 16.0	86.2 ± 19.6	0.945
Arterial Oxygen Saturation (%)	91.4 ± 8.0	91.4 ± 8.8	91.4 ± 6.3	0.996
P/F at presentation	236 ± 86	222 ± 80	260 ± 90	**0.029**

Data are expressed as number (%) or mean ± standard deviation: ARDS = acute respiratory distress syndrome; BMI = body mass index; BSA = body surface area; CABG = coronary artery bypass graft; COPD = chronic obstructive pulmonary disease; ED = emergency department; PCI = percutaneous coronary intervention; P/F = PaO_2_/FiO_2_; statistically significant *p* values are reported in bold.

**Table 2:** Echocardiographic parameters of patients with COVID-19, in the overall population and according to ePLAR values at baseline.

	Overall *n* = 100	High ePLAR *n* = 63	Low ePLAR *n*= 37	*p* Value
LVEDD (mm)	48.1 ± 8.3	46.8 ± 8.3	50.3 ± 8.9	**0.041**
LVEDDi (mm/m^2^)	25.4 ± 4.8	24.1 ± 4.0	27.7 ± 5.2	**<0.0001**
LVESD (mm)	26.6 ± 10.2	23.9 ± 8.5	31.3 ± 11.2	**<0.0001**
LVEDV (mL)	95.3 ± 30.4	95.8 ± 26.4	94.3 ± 36.6	0.816
LVEDVi (mL/m^2^)	50.1 ± 15.9	49.2 ± 11.8	51.6 ± 21.2	0.469
LVESV (mL)	39.8 ± 22.2	37.2 ± 12.9	44.2 ± 32.2	0.130
LVEF (%)	59.4 ± 9.7	61.6 ± 5.8	55.6 ± 13.3	**0.003**
LAVi (mL/m^2^)	27.8 ± 12.4	25.3 ± 11.2	32.1 ± 13.2	**0.008**
E wave velocity (cm/s)	66.7 ± 21.4	59.5 ± 14.4	78.6 ± 25.7	**<0.0001**
A wave velocity (cm/s) *	73.0 ± 20.3	70.1 ± 18.8	79.1 ± 22.3	**0.047**
E/A ratio *	0.9 ± 0.5	0.9 ± 0.3	1.1 ± 0.8	0.075
Mitral lateral e’ velocity (cm/s)	10.0 ± 3.0	10.5 ± 3.0	9.1 ± 2.9	**0.024**
Mitral septal e’ velocity (cm/s)	7.3 ± 2.0	7.0 ± 1.9	7.9 ± 2.2	**0.047**
Mitral medium e’ velocity (cm/s)	9.1 ± 2.6	9.8 ± 2.6	7.9 ± 2.1	**<0.0001**
E/e’	8.0 ± 3.9	6.4 ± 1.9	10.7 ± 4.7	**<0.0001**
RVEDD basal (mm)	36.5 ± 5.1	36.4 ± 4.9	36.7 ± 5.5	0.775
TAPSE (mm)	21.8 ± 4.1	22.8 ± 4.0	20.3 ± 4.0	**0.003**
Tricuspid s’ velocity (cm/s)	14.3 ± 3.7	15.0 ± 3.7	13.0 ± 3.3	**0.016**
Tricuspid e’ velocity (cm/s)	11.4 ± 3.4	11.5 ± 3.6	11.3 ± 3.0	0.849
IVC diameter (mm)	15.0 ± 4.4	14.7 ± 3.8	15.5 ± 5.4	0.399
IVC collapse	92 (92.0)	58 (92.1)	34 (91.9)	0.976
sPAP (mmHg)	32.4 ± 11.2	33.5 ± 11.3	30.6 ± 11.1	0.209
TRV (m/s)	2.4 ± 0.5	2.5 ± 0.5	2.3 ± 0.5	**0.026**
RVSP (mmHg)	27.0 ± 9.8	27.9 ± 9.9	25.6 ± 9.5	0.256
TAPSE/PAPs	0.9 ± 0.5	0.8 ± 0.3	1.0 ± 0.6	**0.024**
Aortic Regurgitation	24 (24.0)	14 (22.2)	10 (27.0)	0.587
Aortic Stenosis	5 (5.0)	2 (3.2)	3 (8.1)	0.274
Mitral regurgitation	74 (74.0)	48 (76.2)	26 (70.3)	0.515
Tricuspid regurgitation	67 (67.0)	45 (71.4)	22 (59.5)	0.219
Pericardial effusion	13 (13.0)	8 (12.7)	5 (13.5)	0.907

* data available on *n* = 91 patients with sinus rhythm (*n* = 62 in high ePLAR group and *n* = 29 in low ePLAR group. Data are expressed as number (%) or mean ± standard deviation: LVEDD = left ventricular end-diastolic diameter; LVEDDi = left ventricular end-diastolic diameter index; LVESD = left ventricular end-systolic diameter; LVEDV = left ventricular end-diastolic volume; LVEDVi = left ventricular end-diastolic volume index; LVESV = left ventricular end-systolic volume; LVEF = left ventricular ejection fraction; LAV = left atrial volume; LAVi = left atrial volume index; RVEDD = right ventricular end-diastolic diameter; TAPSE = tricuspid annular plane systolic excursion; IVC = inferior vena cava; sPAP = systolic pulmonary artery pressure; TRV = tricuspid regurgitation velocity; RVSP = right ventricular systolic pressure; statistically significant *p* values are reported in bold.

**Table 3:** In-hospital adverse events according to ePLAR.

	High ePLAR *n* = 63	Low ePLAR *n* = 37	*p* Value
Acute myocardial infarction	2 (3.2)	2 (5.4)	0.583
Acute heart failure	5 (7.9)	5 (13.5)	0.369
Acute pericarditis	2 (3.2)	2 (5.4)	0.583
Atrio-ventricular block	0 (0.0)	1 (2.7)	0.190
Sustained ventricular tachycardia	1 (1.6)	0 (0.0)	0.441
Ventricular fibrillation	1 (1.6)	2 (5.4)	0.280
Need for ICU	13 (20.6)	5 (13.5)	0.371
Pulmonary embolism	0 (0.0)	2 (5.4)	0.062
Deep vein thrombosis	0 (0.0)	1 (2.7)	0.190
TIA or ischemic stroke	2 (3.2)	0 (0.0)	0.274
Septic shock	6 (9.5)	1 (2.7)	0.197
Acute renal failure	9 (14.3)	5 (13.5)	0.914
In-hospital death	17 (27.0)	4 (10.8)	0.054
MACE	19 (30.2)	8 (21.6)	0.175

Data are expressed as number (%): ePLAR = echocardiographic pulmonary to left atrial ratio; ICU = intensive care unit; MACE = major adverse cardiovascular events (death, acute myocardial infarction, TIA, stroke, or venous thromboembolism); TIA = transient ischemic attack.

**Table 4:** Echocardiographic parameters of survivor and non-survivor patients with COVID-19.

	Survivors *n* = 79	Non-Survivors *n* = 21	*p* Value
LVEDD (mm)	49.0 ± 7.9	44.7 ± 9.1	**0.035**
LVEDDi (mm/m^2^)	25.6 ± 4.7	24.7 ± 5.2	0.439
LVESD (mm)	27.3 ± 10.1	24.2 ± 10.5	0.216
LVEDV (mL)	96.5 ± 32.1	90.1 ± 23.3	0.453
LVEDVi (mL/m^2^)	50.2 ± 17.0	49.5 ± 11.3	0.870
LVESV (mL)	40.1 ± 23.8	38.9 ± 15.4	0.836
LVEF (%)	59.5 ± 10.2	59.1 ± 7.9	0.878
LAVi (ml/m^2^)	28.0 ± 12.8	27.1 ± 10.9	0.778
E wave velocity (cm/s)	66.6 ± 22.7	66.8 ± 15.8	0.977
A wave velocity (cm/s) *	70.1 ± 18.5	84.7 ± 23.7	**0.006**
E/A ratio *	1.0 ± 0.5	0.8 ± 0.2	0.136
Mitral lateral e’ velocity (cm/s)	10.3 ± 3.0	8.8 ± 2.9	0.047
Mitral septal e’ velocity (cm/s)	8.4 ± 2.9	7.3 ± 2.1	0.134
Mitral medium e’ velocity (cm/s)	9.3 ± 2.6	8.1 ± 2.2	**0.049**
E/e’	7.9 ± 4.2	8.5 ± 1.9	0.485
RVEDD basal (mm)	36.4 ± 4.8	36.9 ± 6.3	0.705
TAPSE (mm)	21.8 ± 4.2	22.0 ± 4.2	0.822
Tricuspid s’ velocity (cm/s)	14.5 ± 3.0	13.5 ± 5.5	0.314
Tricuspid e’ velocity (cm/s)	11.5 ± 2.9	11.0 ± 5.1	0.572
IVC diameter (mm)	14.8 ± 4.4	15.8 ± 4.7	0.333
IVC collapse	75 (94.9)	17 (81.0)	**0.036**
sPAP (mmHg)	30.3 ± 9.7	40.6 ± 12.9	**<0.001**
TRV (m/s)	2.4 ± 0.4	2.8 ± 0.5	**<0.001**
RVSP (mmHg)	25.2 ± 8.8	33.7 ± 10.3	**<0.001**
TAPSE/PAPs	1.0 ± 0.5	0.6 ± 0.2	**0.006**
ePLAR (m/s)	0.34 ± 0.15	0.32 ± 0.29	0.754
Aortic Regurgitation	16 (20.0)	8 (38.1)	0.089
Aortic Stenosis	3 (3.8)	2 (9.5)	0.285
Mitral regurgitation	54 (68.3)	20 (95.2)	**0.013**
Mitral stenosis	3 (3.8)	0 (0.0)	0.365
Tricuspid regurgitation	48 (60.8)	19 (90.5)	**0.010**
Pulmonary regurgitation	16 (20.3)	8 (38.1)	0.089
Pericardial effusion	10 (12.7)	3 (14.3)	0.844

* data available on *n* = 91 patients with sinus rhythm (*n* = 73 survivors and *n* = 18 non-survivors); data are expressed as number (%) or mean ± standard deviation: LVEDD = left ventricular end-diastolic diameter; LVEDDi = left ventricular end-diastolic diameter index; LVESD = left ventricular end-systolic diameter; LVEDV = left ventricular end-diastolic volume; LVEDVi = left ventricular end-diastolic volume index; LVESV = left ventricular end-systolic volume; LVEF = left ventricular ejection fraction; LAV = left atrial volume; LAVi = left atrial volume index; RVEDD = right ventricular end-diastolic diameter; TAPSE = tricuspid annular plane systolic excursion; IVC = inferior vena cava; sPAP = systolic pulmonary artery pressure; TRV = tricuspid regurgitation velocity; RVSP = right ventricular systolic pressure; ePLAR = echocardiographic pulmonary to left atrial ratio; statistically significant *p* values are reported in bold.

## Data Availability

The data presented in this study are available on request from the corresponding author. The data are not publicly available due to privacy reason.

## Supplementary Materials

The following supporting information can be downloaded at: https://www.mdpi.com/article/10.3390/diagnostics13020224/s1, Figure S1: Reproducibility assessment between two operators in a random sample of 15 patients (8 from Maggiore della Carità Hospital and 7 patients from SS. Annunziata Hospital) for 4 meaningful echocardiographic parameters: left ventricle ejection fraction (LVEF, panel A), tricuspid regurgitation velocity (panel B), E/e’ ratio (panel C), and ePLAR (panel D). All four parameters resulted in a strong correlation between operators.; Table S1: clinical characteristics of survivor and non-survivor patients with COVID-19.

## References

[1] Klok F.A., Kruip M., van der Meer N.J.M., Arbous M.S., Gommers D., Kant K.M., Kaptein F.H.J., van Paassen J., Stals M.A.M., Huisman M.V. (2020). Incidence of thrombotic complications in critically ill ICU patients with COVID-19. Thromb. Res..

[2] Tang N., Li D., Wang X., Sun Z. (2020). Abnormal coagulation parameters are associated with poor prognosis in patients with novel coronavirus pneumonia. J. Thromb. Haemost..

[3] Gasecka A., Borovac J.A., Guerreiro R.A., Giustozzi M., Parker W., Caldeira D., Chiva-Blanch G. (2021). Thrombotic Complications in Patients with COVID-19: Pathophysiological Mechanisms, Diagnosis, and Treatment. Cardiovasc. Drugs Ther..

[4] Ackermann M., Verleden S.E., Kuehnel M., Haverich A., Welte T., Laenger F., Vanstapel A., Werlein C., Stark H., Tzankov A. (2020). Pulmonary Vascular Endothelialitis, Thrombosis, and Angiogenesis in Covid-19. N. Engl. J. Med..

[5] Carsana L., Sonzogni A., Nasr A., Rossi R.S., Pellegrinelli A., Zerbi P., Rech R., Colombo R., Antinori S., Corbellino M. (2020). Pulmonary post-mortem findings in a series of COVID-19 cases from northern Italy: A two-centre descriptive study. Lancet Infect. Dis..

[6] Pagnesi M., Baldetti L., Beneduce A., Calvo F., Gramegna M., Pazzanese V., Ingallina G., Napolano A., Finazzi R., Ruggeri A. (2020). Pulmonary hypertension and right ventricular involvement in hospitalised patients with COVID-19. Heart.

[7] Li Y., Li H., Zhu S., Xie Y., Wang B., He L., Zhang D., Zhang Y., Yuan H., Wu C. (2020). Prognostic Value of Right Ventricular Longitudinal Strain in Patients With COVID-19. JACC Cardiovasc. Imaging.

[8] Konstantinides S.V., Meyer G., Becattini C., Bueno H., Geersing G.J., Harjola V.P., Huisman M.V., Humbert M., Jennings C.S., Jimenez D. (2020). 2019 ESC Guidelines for the diagnosis and management of acute pulmonary embolism developed in collaboration with the European Respiratory Society (ERS). Eur. Heart J..

[9] Sanchez O., Trinquart L., Planquette B., Couturaud F., Verschuren F., Caille V., Meneveau N., Pacouret G., Roy P.M., Righini M. (2013). Echocardiography and pulmonary embolism severity index have independent prognostic roles in pulmonary embolism. Eur. Respir. J..

[10] Humbert M., Kovacs G., Hoeper M.M., Badagliacca R., Berger R.M.F., Brida M., Carlsen J., Coats A.J.S., Escribano-Subias P., Ferrari P. (2022). 2022 ESC/ERS Guidelines for the diagnosis and treatment of pulmonary hypertension. Eur. Heart J..

[11] Barco S., Mahmoudpour S.H., Planquette B., Sanchez O., Konstantinides S.V., Meyer G. (2019). Prognostic value of right ventricular dysfunction or elevated cardiac biomarkers in patients with low-risk pulmonary embolism: A systematic review and meta-analysis. Eur. Heart J..

[12] Valerio L., Mavromanoli A.C., Barco S., Abele C., Becker D., Bruch L., Ewert R., Faehling M., Fistera D., Gerhardt F. (2022). Chronic thromboembolic pulmonary hypertension and impairment after pulmonary embolism: The FOCUS study. Eur. Heart J..

[13] Lodigiani C., Iapichino G., Carenzo L., Cecconi M., Ferrazzi P., Sebastian T., Kucher N., Studt J.D., Sacco C., Bertuzzi A. (2020). Venous and arterial thromboembolic complications in COVID-19 patients admitted to an academic hospital in Milan, Italy. Thromb. Res..

[14] Scalia G.M., Scalia I.G., Kierle R., Beaumont R., Cross D.B., Feenstra J., Burstow D.J., Fitzgerald B.T., Platts D.G. (2016). ePLAR—The echocardiographic Pulmonary to Left Atrial Ratio—A novel non-invasive parameter to differentiate pre-capillary and post-capillary pulmonary hypertension. Int. J. Cardiol..

[15] Scalia I.G., Scalia W.M., Hunter J., Riha A.Z., Wong D., Celermajer Y., Platts D.G., Fitzgerald B.T., Scalia G.M. (2020). Incremental Value of ePLAR-The Echocardiographic Pulmonary to Left Atrial Ratio in the Assessment of Sub-Massive Pulmonary Emboli. J. Clin. Med..

[16] Lang R.M., Badano L.P., Mor-Avi V., Afilalo J., Armstrong A., Ernande L., Flachskampf F.A., Foster E., Goldstein S.A., Kuznetsova T. (2015). Recommendations for cardiac chamber quantification by echocardiography in adults: An update from the American Society of Echocardiography and the European Association of Cardiovascular Imaging. Eur. Heart J. Cardiovasc. Imaging.

[17] Nagueh S.F., Smiseth O.A., Appleton C.P., Byrd B.F., Dokainish H., Edvardsen T., Flachskampf F.A., Gillebert T.C., Klein A.L., Lancellotti P. (2016). Recommendations for the Evaluation of Left Ventricular Diastolic Function by Echocardiography: An Update from the American Society of Echocardiography and the European Association of Cardiovascular Imaging. Eur. Heart J. Cardiovasc. Imaging.

[18] Bunting K.V., Steeds R.P., Slater L.T., Rogers J.K., Gkoutos G.V., Kotecha D. (2019). A Practical Guide to Assess the Reproducibility of Echocardiographic Measurements. J. Am. Soc. Echocardiogr..

[19] Guzik T.J., Mohiddin S.A., Dimarco A., Patel V., Savvatis K., Marelli-Berg F.M., Madhur M.S., Tomaszewski M., Maffia P., D’Acquisto F. (2020). COVID-19 and the cardiovascular system: Implications for risk assessment, diagnosis, and treatment options. Cardiovasc. Res..

[20] Inciardi R.M., Lupi L., Zaccone G., Italia L., Raffo M., Tomasoni D., Cani D.S., Cerini M., Farina D., Gavazzi E. (2020). Cardiac Involvement in a Patient With Coronavirus Disease 2019 (COVID-19). JAMA Cardiol..

[21] Spinoni E.G., Mennuni M., Rognoni A., Grisafi L., Colombo C., Lio V., Renda G., Foglietta M., Petrilli I., D’Ardes D. (2021). Contribution of Atrial Fibrillation to In-Hospital Mortality in Patients With COVID-19. Circ. Arrhythmia Electrophysiol..

[22] Cummings M.J., Baldwin M.R., Abrams D., Jacobson S.D., Meyer B.J., Balough E.M., Aaron J.G., Claassen J., Rabbani L.E., Hastie J. (2020). Epidemiology, clinical course, and outcomes of critically ill adults with COVID-19 in New York City: A prospective cohort study. Lancet.

[23] Navaratnam A.V., Gray W.K., Day J., Wendon J., Briggs T.W.R. (2021). Patient factors and temporal trends associated with COVID-19 in-hospital mortality in England: An observational study using administrative data. Lancet Respir. Med..

[24] Zhou F., Yu T., Du R., Fan G., Liu Y., Liu Z., Xiang J., Wang Y., Song B., Gu X. (2020). Clinical course and risk factors for mortality of adult inpatients with COVID-19 in Wuhan, China: A retrospective cohort study. Lancet.

[25] Tello K., Axmann J., Ghofrani H.A., Naeije R., Narcin N., Rieth A., Seeger W., Gall H., Richter M.J. (2018). Relevance of the TAPSE/PASP ratio in pulmonary arterial hypertension. Int. J. Cardiol..

[26] Mennuni M.G., Renda G., Grisafi L., Rognoni A., Colombo C., Lio V., Foglietta M., Petrilli I., Pirisi M., Spinoni E. (2021). Clinical outcome with different doses of low-molecular-weight heparin in patients hospitalized for COVID-19. J. Thromb. Thrombolysis.

[27] Investigators A., Investigators A.C.-a., Investigators R.-C., Lawler P.R., Goligher E.C., Berger J.S., Neal M.D., McVerry B.J., Nicolau J.C., Gong M.N. (2021). Therapeutic Anticoagulation with Heparin in Noncritically Ill Patients with Covid-19. N. Engl. J. Med..

[28] Investigators R.-C., Investigators A.C.-a., Investigators A., Goligher E.C., Bradbury C.A., McVerry B.J., Lawler P.R., Berger J.S., Gong M.N., Carrier M. (2021). Therapeutic Anticoagulation with Heparin in Critically Ill Patients with Covid-19. N. Engl. J. Med..

[29] Valerio L., Ferrazzi P., Sacco C., Ruf W., Kucher N., Konstantinides S.V., Barco S., Lodigiani C., Humanitas C.-T.F. (2021). Course of D-Dimer and C-Reactive Protein Levels in Survivors and Nonsurvivors with COVID-19 Pneumonia: A Retrospective Analysis of 577 Patients. Thromb. Haemost..

