# Supplementary material for: Predictive Value of Echocardiographic Pulmonary to Left Atrial Ratio for In-Hospital Death in Patients with COVID-19
Source: Diagnostics (Basel). 2023 Jan 7;13(2):224. doi: 10.3390/diagnostics13020224 (PMC9857775; doi:10.3390/diagnostics13020224)

Supplementary Table S1. Clinical characteristics of survivor and non-survivor patients with COVID-19.

	**Survivors n= 79**	**Non-survivors n= 21**	**P value**
Age (years)	61.8±15.3	76.6±9.0	**<0.001**
Gender female	29 (36.7)	9 (23.7)	0.606
Body weight (Kg)	82.3±18.2	75.3±15.1	0.110
BMI (Kg/m²)	28.4±5.3	27.3±4.9	0.384
BSA (m²)	1.9±0.2	1.8±0.2	0.062
Arterial Hypertension	47 (59.5)	17 (81.0)	0.069
Dyslipidemia	33 (41.8)	8 (38.1)	0.761
Diabetes mellitus	18 (22.8)	10 (47.6)	**0.024**
Smoking	12 (15.2)	4 (19.1)	0.668
Ischemic Heart Disease	9 (11.4)	4 (19.1)	0.354
Non-Ischemic Heart Disease	11 (13.9)	3 (14.3)	0.966
Previous PCI	6 (7.6)	4 (19.1)	0.120
Previous CABG	3 (3.8)	2 (9.5)	0.285
Atrial Fibrillation	10 (12.7)	5 (23.8)	0.203
COPD	10 (12.7)	3 (14.3)	0.844
Active Cancer	5 (6.3)	4 (19.1)	0.070
History of cancer	7 (8.9)	4 (19.1)	0.185
Autoimmune Disease	7 (8.9)	(14.3)	0.461
Chronic renal failure	7 (8.9)	8 (38.1)	**0.001**
Chronic liver disease	0 (0.0)	2 (9.5)	**0.006**
Severe ARDS at ED presentation	21 (26.6)	15 (71.4)	**<0.001**
Systolic Blood Pressure (mmHg)	131.1±18.0	130.3±23.3	0.856
Diastolic Blood Pressure (mmHg)	76.2±12.7	71.8±11.8	0.151
Heart Rate (bpm)	86.7±17.6	85.1±16.4	0.709
Arterial Oxygen Saturation (%)	92.2±6.7	88.3±11.2	**0.047**
P/F at presentation	251.4±80.1	179.0±85.5	**<0.001**

Data are expressed as number (%) or mean ± standard deviation. ARDS = acute respiratory distress syndrome; BMI = body mass index; BSA = body surface area; CABG = coronary artery bypass graft; COPD = chronic obstructive pulmonary disease; ED = emergency department; PCI = percutaneous coronary intervention; P/F = PaO2/FiO2. Statistically significant p values are reported in bold.

Supplementary Figure S1. Reproducibility assessment between two operators in a random sample of 15 patients (8 from Maggiore della Carità Hospital and 7 patients from SS. Annunziata Hospital) for 4 meaningful echocardiographic parameters: left ventricle ejection fraction (LVEF, **panel A**), tricuspid regurgitation velocity (**panel B**), E/e′ ratio (**panel C**), and ePLAR (**panel D**). All four parameters resulted in a strong correlation between operators.

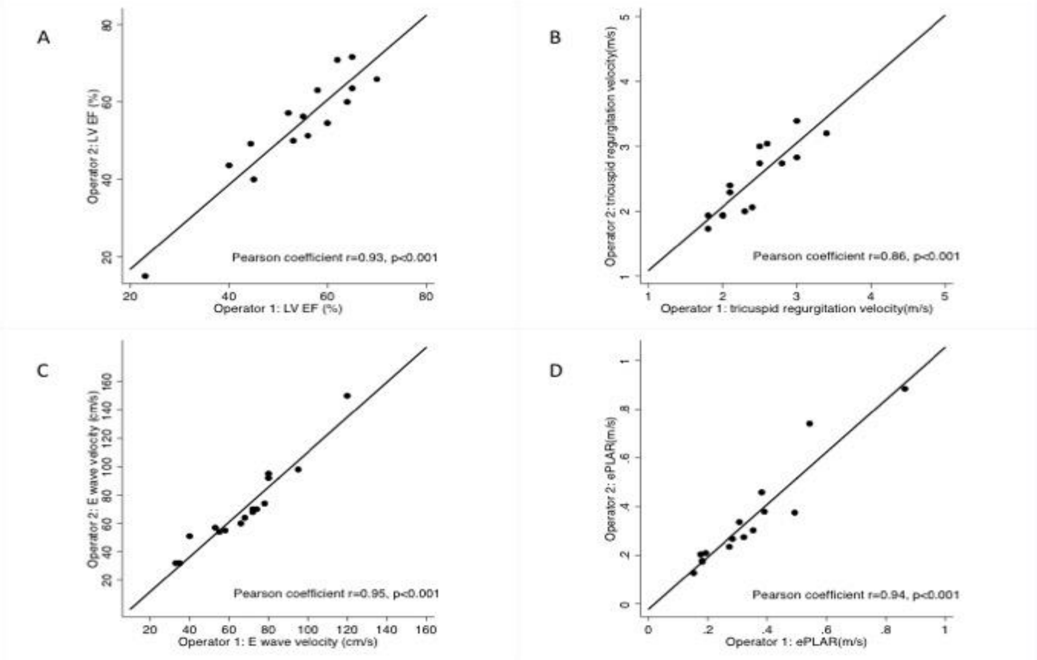

Supplement: Supplementary file 1 [file diagnostics-13-00224-s001.zip › diagnostics-2016102-supplementary.pdf]
